# Randomized clinical trial comparing the effects of sevoflurane and propofol on carbon dioxide embolism during pneumoperitoneum in laparoscopic hepatectomy

**DOI:** 10.18632/oncotarget.15492

**Published:** 2017-02-18

**Authors:** Yu Hong, Yu Xin, Fei Yue, He Qi, Cai Jun

**Affiliations:** ^1^ Department of General Surgery, Sir Run Run Shaw Hospital of Zhejiang University, Hangzhou, Zhejiang, China; ^2^ Department of Anesthesia, Sir Run Run Shaw Hospital of Zhejiang University, Hangzhou, Zhejiang, China; ^3^ Lincoln Christian School, Lincoln, NE, USA

**Keywords:** anesthetics, carbon dioxide embolism, laparoscopic hepatectomy, transesophageal echocardiography

## Abstract

Laparoscopic hepatectomy carries a high risk of gas embolism due to the extensive hepatic transection plane and large hepatic vena cava. Here, we compared the influence of inhaled and intravenous anesthetics on gas embolism during laparoscopic hepatectomy. Fifty patients undergoing laparoscopic hepatectomy were divided into two groups to receive sevoflurane anesthesia (group S, *n* = 25) or intravenous propofol anesthesia (group *p*, *n* = 25). During the operation, gas emboli were detected by transesophageal echocardiography and graded according to their size. Venous CO_2_ emboli were detected in all patients, and the embolism grades did not differ between the two groups. However, the mean embolism episode duration was longer in group S than group P (51.24±23.59 *vs*. 34.00±17.13 sec, *p* < 0.05). At the point of the most severe gas embolism, the PT_CO2_ was higher in group S than group *p* (44.00±4.47 *vs*. 41.36±2.77 mmHg, *p* < 0.05), while the PO_2_/FiO_2_ (450.52±54.08 vs. 503.80±63.18, *p* < 0.05) and pH values (7.35±0.05 *vs*. 7.38±0.02, *p* < 0.05) were lower in group S than group P. Patients with a history of abdominal surgery or liver cirrhosis had higher gas embolism grades. Thus volatile anesthetics may lengthen the duration of embolism episodes and worsen hemodynamics and pulmonary blood gas exchange during surgery.

## INTRODUCTION

In recent years, laparoscopic liver resection has come to be regarded as an effective and safe surgical procedure for liver disease in selected patients. However, this procedure can be complicated by two well-known problems: bleeding and CO_2_ gas embolism (GE). As clinical experience increases and surgical technique improves, bleeding can be minimized or avoided in highly skilled hands. On the other hand, GE is less common but is potentially life-threatening. Aside from the long operative duration, the unique hepatic anatomy full of large-venous and vena- cava can allow gas to enter into the circulatory system through high-pressure insufflations when there are vessels injuries during dissection of the extensive liver parenchyma [1]. Therefore, the risk of GE may be elevated during the procedure of laparoscopic hepatectomy (LH). However, there is no consensus on what conditions cause embolisms or how frequent and serious they are. Most studies have focused on modulating the central venous pressure (CVP) and intraperitoneal pressure to limit this complication [2,3]. However, other risk factors for GE are involved in pneumoperitoneum, such as abdominal surgery history [4], positive end-expiratory pressure [5], liver cirrhosis [6], drug treatment [7], and surgical instrumentation [8]. If the risks of potential hazards are to be minimized, possible parameters influencing the occurrence of GE need to be investigated.

Sevoflurane and propofol are inhaled and intravenous anesthetics, respectively, and are extensively used in clinical anesthesiology. Previous animal studies have demonstrated different anesthetics may influence the severity of experimental venous air emboli, and the filtration capacity of lung would be greatly impaired by the use of halothane [7]. However, these data have never been precisely verified in a clinical setting. In the present study, we assessed the incidence and severity of embolic events when inhaled or intravenous anesthetics were used during LH procedures.

## MATERIALS AND METHODS

After approval was received from the Institutional Review Board for the Protection of Human Subjects at Sir Run Run Shaw Medical Center, Zhejiang University, written informed consent was obtained, and 50 consecutive patients having attempted LH were enrolled in the present study.

Patients with histories of valvular or congenital heart disease, chronic obstructive pulmonary disease, dysphagia, or esophageal disease (e.g., esophageal perforation, hiatal hernia, esophageal stenosis, esophageal varix and so on) were excluded from this study. In addition, patients for whom the surgical approach was changed to open were excluded from the analysis.

A computer-generated 1:1 randomization protocol was used to assign participants to either the sevoflurane or the propofol group for the maintenance of anesthesia. An opaque envelope was opened by the anesthetic assistant who was on duty before the operation contained each subject's random group assignment. The anesthesiologists involved in the study were blinded to the group assignments. Fifty adult patients, ASA I-III, Child-Pugh A and B for attempted LH, were assigned to the sevoflurane (Group S, n=25) or propofol (Group P, n=25) group. All operations were performed by the same attending surgical group (Dr. Cai XJ’).

In Group S, anesthesia was induced with sevoflurane (4-6 vol %), sufentanil (0.6 μg/kg) and rocuronium (0.6 mg/kg), and maintained with sevoflurane (2-3 vol %) and remifentanyl infusion (0.1-0.2 μg/kg^−1^min^−1^). In Group P, anesthesia was induced with propofol (2 mg/kg) and maintained with propofol infusion (4-6 μg/kg^−1^min^−1^) and remifentanyl infusion (0.1-0.2 μg/kg^−1^min^−1^). The anesthesia depth was maintained consistently in the two groups with an entropy index ((E-ENTROPY-01, GE Healthcare, Helsink, Finland)) between 40 and 60. Patients were ventilated with a tidal volume of 8-10 mL/kg at a rate of 8-16 breaths/min with a 50% concentration of O_2_ to maintain an end-tidal CO_2_ (ETco_2_) level of 30-40 mmHg.

After the induction of anesthesia, a transesophageal echocardiography (TEE) probe was inserted via an esophagotomy, and the existence of a patent foramen ovale (PFO) was checked before the surgery. Patients were infused with 1 liter of Lactated Ringer's solution before the operation. During the operation, special attention was paid to the right atrium (RA), right ventricle (RV) and right ventricular outflow tract (RVOT). TEE images were videotaped throughout the surgery. An independent cardiac anesthesiologist categorized GEs into five grades according to Schmandra's classification system [9]: Grade 1, no bubbles in the RA, RV or RVOT; Grade 2, a single gas bubble in the RA, RV or RVOT; Grade 3, gas bubbles filling less than half the diameter of the RA, RV or RVOT; Grade 4, gas bubbles filling more than half the diameter of the RA, RV or RVOT; Grade 5, gas bubbles completely filling the diameter of the RA, RV or RVOT. Severe GE encompassed grades 4 and 5.

Before hepatic dissection, the CVP was adjusted to 8 mmHg and maintained during the resection procedure. Arterial blood pressure, pulse oximetric saturation, peak airway pressure and ETco_2_ levels were monitored throughout the surgery. A sudden decrease in mean arterial blood pressure (MAP) by >30% of the baseline value and/or an acute fall in SPO_2_ were defined as hemodynamic instability. Electrocardiogram changes related to ST elevation and depression (ST elevation >0.1 mV, ST depression <0.05 mV), bradycardia (HR <60 bpm) and tachycardia (HR >100 bpm) were also recorded. A minor resection was defined as a resection of one or two segments, while a major resection was defined as a resection of three or more segments, according to the Brisbane 2000 terminology of liver anatomy and resections [10]. Liver cirrhosis was diagnosed histologically.

Pneumoperitoneum pressure was set at 15 mmHg throughout the operation. The measurements of echocardiographic variables included the grades and durations of the most severe embolism episodes. Arterial blood samples were drawn before the induction, 5 min after the peritoneal insufflation and every 10 min during the liver resection, for the measurement of PO_2_, PCO_2,_ and pH values. The end of the GE period was defined as a bubble-free interval for at least 5 sec. The blood gas was measured at the closet point to the most severe GE.

### Statistical analyses

Statistical analyses were performed with SPSS 20.0. Data are presented as the mean±SD. Patients’ demographic and surgical characteristics were compared through a chi-square test. Differences in the incidence of GE between the two groups were analyzed by the Mann-Whitney U test. The embolism durations and the MAP, HR, CVP, ET_CO2,_ PO_2_/FiO_2_ and pH values at the severe periods during surgery were compared through Student's t-test. A p value <0.05 was considered statistically significant.

## RESULTS

Fifty patients were enrolled in the present study, but one patient in the S group was excluded from the analysis due to a change of the laparoscopy to an open procedure. The demographic and surgical characteristics were similar in the two groups (Table 1). One patient in the S group had a PFO, and none of patient in the present study had a history of cirrhosis and abdominal surgery.

**Table 1:** Demographic and surgical characteristics of the two groups of patients

	Group S (*N*= 24)	Group *P* (*N*= 25)	*P* value
Age (y)	52.57±14.23	54.07±13.31	0.772
Gender Male/Female	10/14	9/16	0.773
BMI (kg/m^2^)	23.40±2.89	23.83±4.24	0.673
MAP (mmHg)	85.63±10.01	88.20±7.53	0.313
ASA (I /II /III)	5/19/0	3/20/2	0.434
Child-Plug (A/B)	14/10	14/11	0.774
Cirrhosis (Y/N)	6/18	8/17	0.754
PFO (Y/N)	1/23	0/25	0.312
Operative time (min)	138.00±67.64	141.00±61.96	0.871
CVP (mmH2O)	6.86±1.51	6.73±1.39	0.820
Pneumoperitoneal time (min)	126.79±70.07	99.33±38.59	0.198
Liver section time (min)	18.58±11.32	18.68±9.25	0.973
Blood loss (mL)	250.00±257.80	384.00±386.15	0.155
Fluid (mL) Crystal Colloid	1278.00±429.17 850.00±375.00	1340.00±624.50 947.92±255.16	0.684 0.293
Arrhythmias	2	1	0.598
Surgical type Major resection Minor resection	10 14	12 13	0.776
Diagnosis Hepatolith Hemangioma Carcinoma Focal nodular hyperplasia	5 2 15 2	4 7 14 0	

The values are mean ± SD

**Figure 1:**
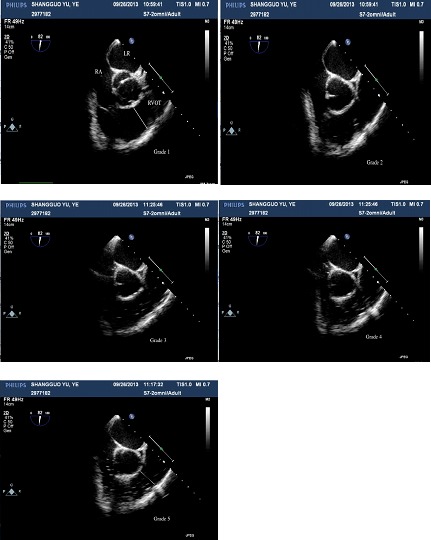
Five-grade embolism detected with TEE in one patient RA: right atrium, LA: left atrium, RVOT: right ventricular outflow tract.

GEs occurred in 100% of the patients. As shown in Figure 1, five-grade embolism was detected by TEE during LH. The distributions of GE grades were similar in the two groups (Table 2). The frequencies of severe GEs (stage 4-5) were 54.2% and 76.0% in the P and S groups, respectively, but this difference was not statistically significant. A total of 20.8% of patients (n=5) in the S group and 16% of patients (n=4) in the P group experienced a severe GE of stage 5.

**Table 2:** Embolism grades and durations of the two groups

	Group S (*N*=24)	Group P (*N*= 25)	*P* value
TEE Grade (1-5)	0 / 3/ 8/ 8/ 5	0/ 0/ 6/ 15/ 4	0.138
TEE Grade (4-5)	13/24	19/25	0.108
Embolism Time (sec)	51.24±23.59	34.00±17.13	0.005*

The values are mean ± SD

*There was a significant difference between the two groups

The PT_CO2_ at the point of the occurrence of the most severe GE was higher in the S group than in the P group (44.00±4.47 mmHg, p<0.05). The MAP and pH values were significantly lower in the S group than in the P group (MAP: 73.14±8.73 vs. 81.73±7.93 mmHg, p<0.05; pH: 7.35±0.05 vs. 7.38±0.02, p<0.05). PO_2_ and PO_2_/FiO_2_ were much lower in the S group than in the P group (PO_2_: 272.52±32.54 vs. 317.84 ± 45.22 mmHg, p<0.05; PO_2_/FiO_2_: 450.52±54.08 vs. 503.80±63.18, p<0.05). With respect to the HR, CVP, ETco_2_, and increase in ETco_2,_ no differences were observed between the two groups (Table 3). In addition, there were no differences in operative blood loss or fluid administration between the groups.

**Table 3:** BP, HR, CVP, ETco_2_ and blood gas values at the point of the most severe gas embolism in the two groups

	Group S (*N* =24)	Group P (*N* =25)	*P* value
MAP (mmHg)	73.14±8.73	81.73±7.93	0.010*
HR (beats/min)	63.29±6.41	65.33±6.84	0.414
CVP (mmH_2_O)	7.00±1.58	6.76±1.45	0.579
ETco_2_^a^ (mmHg)	35.16±1.21	35.28±1.99	0.798
ETco_2_^b^ (mmHg)	43.48±4.25	41.80±2.69	0.102
ETco_2_^b-a^ (mmHg)	8.32±4.46	6.52±2.45	0.083
PT_CO2_ (mmHg)	44.00±4.47	41.36±2.77	0.015*
PO_2_ (mmHg)	272.52±32.54	317.84±45.22	0.000*
PO_2_/FiO_2_	450.52±54.08	503.80±63.18	0.002*
PH	7.35±0.05	7.38±0.02	0.007*

* There were significant differences between the two groups

ETco_2_^a^: end-tidal carbon dioxide before the pneumoperitoneum

ETco_2_^b^: End- tidal carbon dioxide at the point of the most severe GE

ETco_2_^b-a^: ETco_2_^b^-ETco_2_^a^

The mean duration of the most severe GE was much longer in S group than in the P group (51.24±23.59 sec vs. 34.67±17.13 sec, p=0.033).

**Figure 2:**
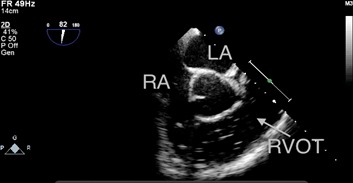
Bubbles filling the RA and RVOT were detected by TEE in one patient in the S group during hepatic parenchymal resection The patient exhibited notable hypotension and a decrease in end-tidal carbon dioxide (from 37 mmHg to 12 mmHg). RA: right atrium, LA: left atrium, RVOT: right ventricular outflow tract.

One patient in the S group experienced a severe GE (grade 5) with low blood pressure and ETco_2_ values, and bubbles filling the RA and RVOT, as detected by TEE (Figure 2 and Video 1). However, the patient was discharged from the hospital without any complications.

The GE grades in patients undergoing LH were higher in those with liver cirrhosis than in those without, and the durations of severe GE were longer in patients undergoing major hepatectomy than in those undergoing minor hepatectomy (Table 4).

**Table 4:** Embolism grade and severe embolism duration in patients with cirrhosis, abdominal surgery history, or major/minor hepatectomy

Variables	TEE Grade (4-5)	*P* Value	Embolism Time (sec)	*P* Value
Cirrhosis		0.01*		0.096
Yes	13 (14)		51.00±23.89	
No	19 (35)		39.36±20.93	
Abdominal history		0.031*		0.080
Yes	17 (18)		38.50±22.39	
No	18 (31)		49.94±20.38	
Major hepatectomy	16 (21)	0.229	50.00±23.19	0.036*
Minor hepatectomy	16 (28)		36.82±19.89	

* There were significant differences between the two groups

## DISCUSSION

The keys to performing LH safely are minimizing bleeding and avoiding CO_2_ GE. Methods such as the Pringle maneuver, along with the available technology, regional occlusion of inflow and outflow, etc., are known to be useful for reducing intraoperative bleeding [11, 12]. Recently, more attention has been paid to air embolism under pneumoperitoneum [3, 5, 9]. The clinical presentation of GE may be asymptomatic or may lead to arrhythmia, cardiovascular collapse, and even death [13]. And changes in the systemic circulation, such as changes in the MAP or pulse rate, are not specific or sensitive for GE, except in the very late, fatal phase. Therefore, clinicians must be aware of this silent but dangerous entity. However, there is no consensus yet on how frequently GEs occur or how they can be prevented. Most previous studies have focused on modulating the intraperitoneal pressure and CVP during laparoscopic surgery [3, 13]. If the risks of the potential hazards are to be minimized, additional parameters need to be considered, such as the learning curves of the operators, duration of pneumoperitoneum, types of resection and instruments, and use of positive end-expiratory pressure.

In the present study, we investigated the influence of different types of anesthetics on the occurrence and duration of GE during LH. TEE, the most sensitive method [14], was applied. The results demonstrated that the incidence of CO_2_ emboli during LH could be as high as 100%. With the exception of one patient, even high-grade GE did not lead to severe hemodynamic instability. In addition, we found that patients with liver cirrhosis or abdominal surgery history experienced more severe GEs than their respective counterparts.

We also found that GEs took place throughout all phases of the dissection, but predominantly occurred during two distinct periods: the liver capsule opening, and the deeper plane of parenchymal transection. Four to fifteen episodes of embolism per patient were detected during CO_2_ pneumoperitoneum, and more than 80% of these embolisms occurred during parenchymal resection. It seems that the passage of insufflants along pressure gradients into large open venous sinuses during deeper liver parenchymal resection makes it easy for GE to occur. The mechanisms promoting the occurrence of GE at the point when the capsule is opened are still not clear. An explanation might be that the intact and dense liver capsule is the first barrier against GE during pneumoperitoneum.

We compared not only the grades of GE events, but also the durations of the most severe GE occurrences during the procedure between the two groups. Our results indicated that the duration of the most severe GE episode was much longer in the sevoflurane group than in the propofol group. Furthermore, the patients had significantly higher PT_CO2_ values and lower MAP, PO_2_/FiO_2_ and pH values in the S group than in the P group. The significantly lower MAP was caused by reduced cardiac output due to the longer retention of bubbles in the RVOT. Above all, the longer duration of severe GE in the S group influenced the hemodynamics and pulmonary blood gas change much more seriously than that in the P group.

The pulmonary circulation and alveolar interface are effective reservoirs for intravascular gas dissipation. Once bubbles enter the lungs, elimination occurs primarily via the alveoli, while part of the gas is dissolved in the blood and adjacent tissues, though to a lesser degree. The physiologic filtering capacity of the lungs is easily overwhelmed when a certain amount of GE is exceeded, especially in case of inhaled anesthetics [15, 16]. Thus, it was not surprising that more time was needed for embolism absorption, resulting in serious respiratory gas exchange and hemodynamic disturbances in the S Group. The mechanism whereby different anesthetic drugs impair the gas filtering capacity of the lungs remains elusive. In a previous study, Kaze et al. reported that compared to intravenous anesthetics, inhaled drugs decreased the diameter of the pulmonary vessels, which lead to a more serious gas embolization. [17]. Yahagi et al. determined that intravenous anesthetics such as fentanyl or ketamine increased the mean pulmonary artery pressure and pulmonary vascular resistance in dogs [7]. This may have been due to changes in pulmonary blood flow at the level of the capillaries, shunt vessels or arterioles. A further study revealed that continuous insufflation of one type of gas (such as sevoflurane) might lead to the diffusion of other types of gas (mainly oxygen and carbon dioxide) into the carbon dioxide emboli [18], thus increasing the time required for embolic absorption. Therefore, we speculated that impairment of the pulmonary filtration capacity by inhaled anesthetics inhabited the passage of bubbles across the lungs by reducing the pulmonary vascular tone, and that the effects of the emboli were enhanced by different gases. Moreover, there is increasing evidence to support the notion that an extended absorption time may be responsible for permanent injuries and deaths after carbon dioxide embolisms [19, 20].

One patient in the S Group exhibited cardiorespiratory instability during the hepatic parenchymal resection. The reason for this may have been be that the entry of CO_2_ through the open middle hepatic vein in a short time led to a state of circulation failure, with a notable reduction in ETco_2_ (from 37 mmHg to 12 mmHg) and subsequent lung hypoperfusion. The TEE monitor displayed the amount of gas accumulating in the RVOT (Figure 2 and Video 1). The transient accumulation of gas in the pulmonary artery caused an “air lock” of the right heart cavities and reduced inflow into the left side of the heart, which could have induced acute hypotension or even cardiac arrest. Overall, the clinical influence of GE depends on the balance between the volume of gas entering the circulation and the amount of gas that is removed [21].

Interestingly, we observed that high-grade GE occurred in patients with cirrhosis and abdominal surgery history. Peritoneal adhesions and further vascular injury after a previous surgery might have facilitated the entry of CO_2_ into the circulation system during pneumoperitoneum [4]. The reason that patients with cirrhosis were more prone to severe GE is still unclear. We propose that the hepatic venous sinus and hepatic vein may have been pressed by hepatic cirrhosis nodules, thus impairing the buffering capacity of the liver sinus for gas absorption. Moreover, fibrotic parenchyma caused by atrophic change and chronic inflammation may have increased the operative difficulty and the risk of GE. The incidence of intacardiac right to left shunt was reported to reach 47% in patients of cirrhosis compared to only 20% in the normal population ^6^. In addition, direct intrapulmonary artery venous shunting is common in chronic liver cirrhosis patients. Although there were no patients with adverse neurologic outcomes in this study, neurological damage due to a paradoxical GE is more likely to occur in cirrhosis patients. Therefore, clinicians must be aware of this silent but dangerous event in advanced chronic liver disease patients.

In an effort to reduce the effects of other factors that could have influenced the incidence of air embolism, we adopted the methodology of randomization. We previously reported that CVP decreased 37% when patients were in the reverse Trendelenburg position during LH [22]. Considering that CVP can be influenced by many factors, we infused patients with 1 liter of Lactated Ringer's solution to increase the CVP during anesthetic induction, and maintained the CVP at the level of 6-7 mmHg while patients were in the Trendelenburg position during the operation. Also, in order to avoid fluid bubbles caused by the administration of fluid and agents, we reduced the fluid infusion rate to a low rate (50 mL/min) and stopped the TEE video recording when a bolus of medication was given. Additionally, the surgeons preforming the procedure were all experienced, according to the learning curve of LH in our center [23].

The incidence of GE in patients undergoing LH was 100%. No single surveyed factor can explain the phenomenon of GE, and the reaction to GE is also hard to predict, although it is likely to depend on the gas volume, entrance rate, and so on. We found that even high-grade GE was clinically insignificant. As patients with preexisting cardiopulmonary disease were excluded from our study, and the operations were performed by experienced surgeons, our results strongly suggest that PFO and liver cirrhosis are possible risk factors for paradoxical air emboli. Therefore, additional monitoring or preparation for early intervention should be performed for patients with major hepatic resection, cardiopulmonary diseases, abdominal surgery history, or liver cirrhosis. Moreover, total intravenous anesthetics are preferred for these patients, and TEE monitoring is valuable for reducing the occurrence of fatal GE.

In conclusion, multiple factors influence GE during LH. Careful monitoring, suitable drug treatment and skilled surgeons are important for minimizing the severity and frequency of GE, especially for patients with abdominal surgery history or liver cirrhosis.

### Limitations

One potential limitation of this study was that we did not measure the mean pulmonary artery pressure or pulmonary vascular resistance to detect the mechanism whereby the filtration of lung was impaired. Another limitation was the small number of the cases; a further study with a larger volume would be needed to validate our conclusions.

## Abbreviations

GE: Gas embolism
LH: Laparoscopic hepatectomy
CVP: Central venous pressure
TEE: Transesophageal echocardiography
PFO: Patent foramen ovale
RA: Right atrium
RV: Right ventricle
RVOT: Right ventricular outflow tract
MAP: Mean arterial pressure
